# Diagnostic Utility of N-Terminal Pro-B-Type Natriuretic Peptide in Identifying Atrial Fibrillation Post-Cryptogenic Stroke: A Systematic Review and Meta-Analysis

**DOI:** 10.3390/pathophysiology31030024

**Published:** 2024-06-30

**Authors:** Jay Patel, Sonu M. M. Bhaskar

**Affiliations:** 1 Global Health Neurology Laboratory, Sydney, NSW 2150, Australia; z5264487@ad.unsw.edu.au; 2 UNSW Medicine and Health, University of New South Wales (UNSW), South West Sydney Clinical Campuses, Sydney, NSW 2170, Australia; 3 Department of Neurology & Neurophysiology, Liverpool Hospital, South West Sydney Local Health District, Liverpool, NSW 2170, Australia; 4 NSW Brain Clot Bank, NSW Health Pathology, Sydney, NSW 2170, Australia; 5 National Cerebral and Cardiovascular Center (NCVC), Department of Neurology, Division of Cerebrovascular Medicine and Neurology, Suita 564-8565, Osaka, Japan

**Keywords:** atrial fibrillation, stroke, biomarker, BNP, NT-proBNP, cryptogenic

## Abstract

Background: Atrial fibrillation (AF) significantly contributes to acute ischemic stroke, with undetected AF being a common culprit in cryptogenic strokes. N-terminal pro-B-type natriuretic peptide (NT-proBNP), indicative of myocardial stress, has been proposed as a biomarker for AF detection, aiding in the selection of patients for extended cardiac monitoring. However, the diagnostic accuracy of NT-proBNP remains uncertain. Methods: We conducted a meta-analysis to evaluate the diagnostic accuracy of NT-proBNP in detecting AF among cryptogenic stroke patients. A comprehensive literature search was conducted across PubMed, Embase, and Cochrane databases to identify relevant studies. Studies reporting NT-proBNP levels in stroke patients and data on the proportion of patients with AF above a specified cut-off were included. Meta-analyses were performed using the midas command in STATA. Results: Seven studies encompassing 2171 patients were included in the analysis, of which five studies contained cohorts with cryptogenic strokes. Among patients with cryptogenic stroke, NT-proBNP demonstrated a diagnostic accuracy of 80% (Area Under the Receiver Operating Curve 0.80 [95% CI 0.76–0.83]), with a sensitivity of 81% (95% CI 0.68–0.89) and a specificity of 68% (95% CI 0.60–0.75). Conclusion: Our meta-analysis indicates that NT-proBNP exhibits a good-to-very-good diagnostic accuracy for detecting AF in patients with cryptogenic stroke. These findings suggest potential implications for utilizing NT-proBNP in guiding the selection of patients for prolonged cardiac monitoring, thereby aiding in the management of cryptogenic stroke cases.

## 1. Introduction

Atrial fibrillation (AF) stands as a prevalent cardiac arrhythmia globally, significantly contributing to both morbidity and mortality [1]. It manifests as an irregularly irregular heart rhythm, categorized as paroxysmal, persistent, or permanent [2]. The pathophysiology of AF is multifaceted, involving cardiac remodeling leading to structural and electrical changes [3]. Additionally, AF correlates with various cardiovascular conditions, such as hypertension, valvular heart disease, and coronary heart disease, alongside risk factors like diabetes and smoking [3,4].

Among the primary concerns in AF patients lies the risk of acute ischemic stroke, attributed to vascular stasis potentially culminating in left atrial thrombus formation and subsequent embolization to the brain [5]. Ischemic stroke is a major cause of disability and is a prominent contributor to the global burden of disease [6]. As part of routine assessment, ischemic stroke patients undergo electrocardiography (ECG) to detect underlying AF [7]. In cases where AF is identified, oral anticoagulant therapy is recommended to mitigate stroke recurrence [8]. This recommendation holds particular relevance for patients with cryptogenic stroke, a subset of ischemic stroke where the stroke’s cause remains unidentified despite investigation [9]. In this subset, studies utilizing implantable cardiac monitors (ICMs) have revealed AF detection rates ranging from 26 to 36% after at least a year of monitoring [10,11]. However, the logistical and economic challenges of long-term ICM monitoring pose limitations in its widespread application among cryptogenic stroke patients [12]. Consequently, there is considerable interest in biomarkers capable of predicting AF post-stroke, facilitating patient stratification for prolonged cardiac monitoring [13].

One such potential biomarker is the N-terminal pro-B-type natriuretic peptide (NT-proBNP), released by atrial and ventricular cardiomyocytes in response to various stimuli such as myocardial wall stress, ischemia, and volume overload [14,15]. Upon cleavage into the biologically active B-type natriuretic peptide (BNP), it induces vasodilation, increased fluid excretion, and inhibition of the renin-angiotensin-aldosterone system (RAAS) [14]. While NT-proBNP and BNP biomarkers are currently employed to assess heart failure, particularly in emergency settings, to rule out cardiac cause of dyspnea [14], there is growing interest in their utility for AF diagnosis, especially in the context of stroke [16].

Though several studies have explored the efficacy of these biomarkers in detecting AF post-stroke [13,17,18,19,20,21,22], results have been variable, leaving the true accuracy uncertain. A meta-analysis presents a valuable opportunity to combine these studies, thereby examining diagnostic accuracy comprehensively. Given NT-proBNP’s longer half-life compared to BNP (120 min vs. 20 min) [14,15], coupled with reported higher diagnostic accuracy for AF [16], our study focuses on NT-proBNP.

This meta-analysis aims to investigate the diagnostic accuracy of NT-proBNP in detecting AF following stroke, particularly in cryptogenic stroke patients. Diagnostic accuracy parameters such as sensitivity, specificity, and the Area Under the Receiver Operating Curve (AUROC) will be analyzed. Additionally, we will compare NT-proBNP accuracy in cohorts where the stroke’s etiology is known, serving as a comparative measure. Our primary research questions are the following:

1. What is the diagnostic accuracy of NT-proBNP in detecting AF in patients with cryptogenic stroke? (As a secondary aim, we will investigate NT-proBNP accuracy in cohorts where the stroke’s etiology is established, thus formulating our secondary research question).
2. What is the diagnostic accuracy of NT-proBNP in detecting AF in patients with stroke of known etiology?

## 2. Methods

### 2.1. Literature Search: Identification and Selection of Studies

**Figure 1:**
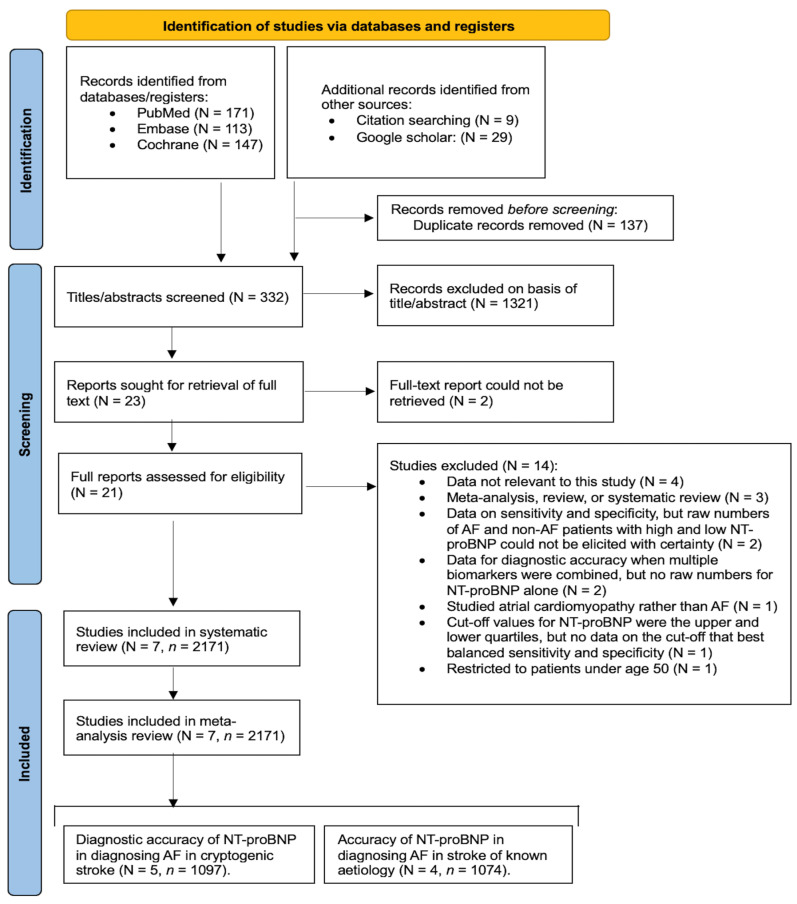
PRISMA flowchart depicting the process of study identification, screening, and inclusion. Abbreviations: N = number of studies, *n* = total number of patients, AF = atrial fibrillation, NT-proBNP = N-terminal pro-B-type natriuretic peptide.

We selected studies from the databases of PubMed, Embase, and Cochrane. We obtained additional studies from Google Scholar and conducted hand searching within the references of other studies. The PRISMA flowchart illustrates the process of study identification and screening, providing the number of studies at each stage and the reasons for study exclusion (Figure 1). Details of the keywords and filters used can be found in Supplemental Information (Search Strategy). We conducted this study following the Preferred Reporting Items for Systematic Reviews and Meta-Analyses (PRISMA) guidelines and adhered to the Meta-analysis of Observational Studies in Epidemiology (MOOSE) checklist (Supplemental Tables S1 and S2), as well as the Standards for Reporting Diagnostic Accuracy Studies (STARD) 2015 guidelines (Supplementary Table S3). This study was registered in Open Science, registration number “pn27u” (https://osf.io/pn27u/ (accessed on 24 March 2024).

### 2.2. Inclusion and Exclusion Criteria

The study’s inclusion criteria encompassed (1) patients diagnosed with acute ischemic stroke; (2) patients aged 18 years and above; (3) a defined cut-off value for high NT-proBNP levels; (4) availability of data on the number of AF and non-AF patients with NT-proBNP levels above and below the cut-off; and (5) studies with robust methodological design, requiring a minimum sample size of 20 patients. Studies meeting these criteria were included, while those failing to meet these conditions were excluded. Criteria for exclusion included (1) animal studies; (2) inaccessible studies where the complete report could not be retrieved; (3) studies published in languages other than English; and (4) studies sourced from the same database as another included study in the meta-analysis.

### 2.3. Data Extraction

Data extraction involved screening the titles and abstracts of identified studies using EndNote 20.6 (Clarivate, Philadelphia, PA, USA). Studies that did not align with the specified inclusion and exclusion criteria were excluded at this stage. From studies passing this initial screening, full texts were retrieved when feasible, and eligibility was further assessed. This screening process was conducted independently by two authors, with any disagreements resolved through discussion until consensus was achieved.

From the studies included in the meta-analysis, data were extracted and organized into a comprehensive spreadsheet. This spreadsheet documented the following information: (1) study characteristics (author, title, year, country, study type); (2) stroke cohort (cryptogenic stroke or stroke of known origin); (3) basic patient characteristics (number of means, mean age, proportion of male sex); (4) prevalence of comorbidities (hypertension, diabetes, dyslipidemia, smoking); (5) NT-proBNP cut-off level; and (6) number of AF and non-AF patients with NT-proBNP levels above and below the cut-off, necessary for sensitivity and specificity calculations. In instances where age was reported using the median and interquartile range (with a clearly stated value for the upper and lower quartile), the mean age and standard deviation were estimated using the method described by Wan et al. [23].

### 2.4. Methodological Quality Assessment of Included Studies

To assess the methodological quality of the studies in the meta-analysis, we evaluated each study using the criteria outlined in the modified Jadad scale [24]. This included considering factors such as randomization and blinding, as well as clear descriptions of the inclusion and exclusion criteria and the statistical analysis method. The complete criteria and evaluation for each study are detailed in Supplementary Table S4. Additionally, we investigated the potential for funding bias by examining the presence of industry funding and conflicts of interest among authors. The evaluation of funding bias is also provided in Supplementary Table S4.

### 2.5. Statistical Analyses

We utilized STATA (Version 13.0, StataCorp, College Station, TX, USA) for statistical analyses. We analyzed the diagnostic accuracy of NT-proBNP in identifying AF following stroke through meta-analyses. These meta-analyses were conducted when at least four studies were available for a given analysis. We employed the *midas* package in STATA for these analyses. The required data for this command included (1) true positives or “tp” (number of patients with NT-proBNP levels above the cut-off diagnosed with AF); (2) false positives for “fp” (number of patients with NT-proBNP levels above the cut-off but without AF); (3) false negatives or “fn” (number of patients with NT-proBNP levels below the cut-off but with AF); and (4) true negatives or “tn” (number of patients with NT-proBNP levels below the cut-off without AF).

With these data, the *midas* package in STATA calculated the sensitivity and specificity of each study. It also combined these data to provide pooled sensitivity and specificity and constructed forest plots. We generated summary receiver operating characteristic (SROC) curves and assessed the AUROC as an overall measure of diagnostic accuracy. A well-established definition was used to describe the relationship between AUROC, and diagnostic accuracy, whereby an AUROC of 0.9–1 represents excellent diagnostic accuracy, an AUROC of 0.8–0.9 is very good diagnostic accuracy, an AUROC of 0.7–0.8 is good, an AUROC of 0.6–0.7 is sufficient, an AUROC 0.5–0.6 is bad diagnostic accuracy, and an AUROC less than 0.5 suggests that a diagnostic accuracy test is not useful [25]. The STATA output additionally provided information regarding positive and negative likelihood ratios alongside graphs representing the likelihood matrix. For all analyses in this study, we considered *p*-values less than 0.05 as statistically significant. Heterogeneity was assessed using the I^2^ statistic, where values of 0–40%, 30–60%, 50–90%, and 75–100% represented low, moderate, substantial, and considerable heterogeneity, respectively [26].

## 3. Results

### 3.1. Results of the Search

The search across PubMed, Embase, and Cochrane databases yielded 431 studies, as outlined in the PRISMA flowchart (Figure 1). Additionally, 29 studies were sourced from Google Scholar, and 9 were identified through manual citation searching within previous studies. Following the removal of duplicates, titles, and abstracts, 332 studies were screened using EndNote. Among these, 23 were found to approximately align with our study criteria, and full-text reports of 21 studies were obtained. These 21 studies underwent rigorous screening to assess their alignment with our study’s inclusion and exclusion criteria. Fourteen studies were excluded, with reasons detailed in the PRISMA flowchart (Figure 1). Consequently, seven studies met the criteria for inclusion in our systematic review and meta-analysis.

### 3.2. Description of the Included Studies

This meta-analysis comprised seven studies [13,17,18,19,20,21,22], encompassing a total of 2171 patients. Of these, five studies [13,17,19,20,21] focused on assessing the diagnostic accuracy of NT-proBNP in detecting AF among patients with cryptogenic stroke, while four studies [13,18,19,22] evaluated its diagnostic accuracy in patients with stroke of known origin. Table 1 presents the baseline characteristics of the studies within the meta-analysis, encompassing study type, country, sex distribution, mean age, and the AF detection method employed in each study. Table 2 provides additional information on the prevalence of various comorbidities within each cohort. A summary of the sensitivities and specificities observed in each individual study cohort is provided in Table 3. Additionally, Table 4 provides a detailed overview of the outcomes derived from the diagnostic accuracy meta-analyses conducted for cohorts of patients with cryptogenic stroke and those with stroke of known origin.

### 3.3. Diagnostic Accuracy of NT-proBNP in Detecting Atrial Fibrillation in Patients with Cryptogenic Stroke

**Figure 2:**
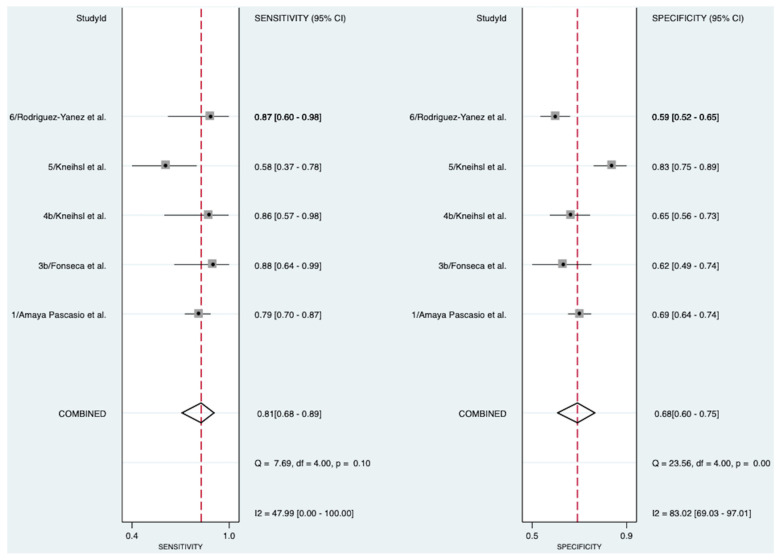
Pooled diagnostic sensitivity and specificity of NT-proBNP in detecting AF in patients with cryptogenic stroke [13,17,19,20,21]. Abbreviations: NT-proBNP = N-terminal pro-B-type natriuretic peptide, CI = confidence interval.

Five studies investigated the diagnostic accuracy of NT-proBNP in detecting AF in patients with cryptogenic stroke [13,17,19,20,21], encompassing 1097 patients (Figure 2). The meta-analysis revealed a good-to-very-good diagnostic accuracy of 80% (AUROC 0.80 [95% CI 0.76–0.83]). NT-proBNP demonstrated a pooled diagnostic sensitivity of 81% (Sensitivity 0.81 [95% CI 0.68–0.89]) and a pooled diagnostic specificity of 68% (Specificity 0.68 [95% CI 0.60–0.75]). The SROC curve can be found in Supplementary Figure S1. Moderate heterogeneity was observed for the test of sensitivity (I^2^ = 47.99, [95% CI 0.00–100.00]), and substantial-to-considerable heterogeneity for the test of specificity (I^2^ = 83.02, [95% CI 69.03–97.01]).

### 3.4. Diagnostic Accuracy of NT-proBNP in Detecting Atrial Fibrillation in Patients with Stroke of Known Etiology

**Figure 3:**
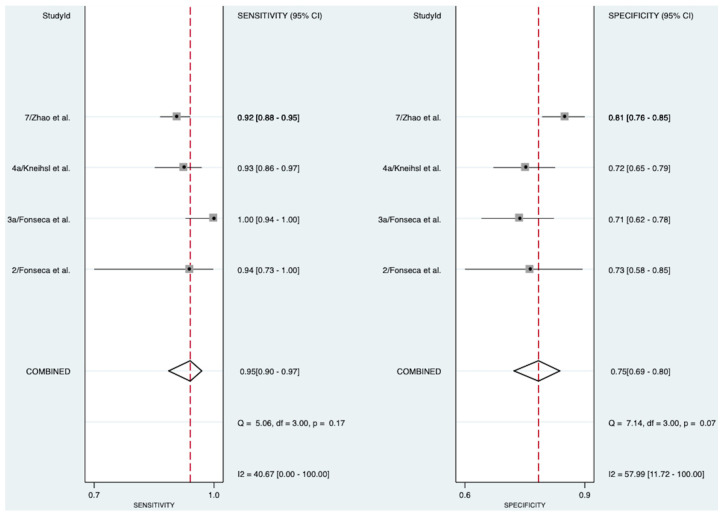
Pooled diagnostic sensitivity and specificity of NT-proBNP in detecting AF in patients with stroke of known etiology [13,18,19,22]. Abbreviations: NT-proBNP = N-terminal pro-B-type natriuretic peptide, CI = confidence interval.

In patients with stroke of known etiology, four studies involving 1074 patients (Figure 3) explored the diagnostic accuracy of NT-proBNP in detecting AF [13,18,19,22]. The meta-analysis indicated an excellent diagnostic accuracy of 91% (AUROC 0.91 [95% CI 0.88–0.93]). NT-proBNP exhibited a pooled diagnostic sensitivity of 95% (Sensitivity 0.95 [95% CI 0.90–0.97]) and a pooled diagnostic specificity of 75% (Specificity 0.75 [95% CI 0.69–0.80]). The SROC curve, likelihood ratio matrix, and goodness of fit plots can be found in Supplementary Figures S1, S2, and S3, respectively. Moderate heterogeneity was observed for the test of sensitivity (I^2^ = 40.67, [95% CI 0.00–100.00]), and moderate-to-substantial heterogeneity for the test of specificity (I^2^ = 57.99, [95% CI 11.72–100.00]).

## 4. Discussion

Pooling the current data, our study revealed that NT proBNP had a good-to-very-good diagnostic accuracy for detecting AF following cryptogenic stroke. This may have promising applications in stratifying patients for cardiac monitoring. The sensitivity and specificity of NT-proBNP for AF detection were comparatively higher in patients with stroke of known etiology, possibly due to variations in AF subtypes among different patient groups.

Our investigation builds upon the findings of a previous meta-analysis by Zhang et al. [16], which reported NT-proBNP’s sensitivity at 91% and specificity at 77% in detecting covert AF following stroke. However, our meta-analysis on cryptogenic stroke yielded lower sensitivity and specificity values, likely attributable to differences in the composition of included studies. Notably, our study incorporated three additional datasets concerning cryptogenic stroke [13,17,20], and one dataset from Fonseca et al. [19], previously analyzed within Zhang et al. [16]), was reassigned to the section focusing on stroke of known etiology. Additionally, we excluded the study by Sanak et al. [29] due to its restriction to patients under 50. Although investigating biomarker accuracy in younger patients holds importance, our inclusion criteria aimed to encompass all adults aged 18 and above without imposing additional age limits.

Our study contributes to the expanding body of evidence suggesting NT-proBNP holds potential as a biomarker for AF following cryptogenic stroke [16,19,30]. Offering a straightforward test, NT-proBNP empowers clinicians to evaluate each cryptogenic stroke patient’s likelihood of having underlying AF more precisely [21]. This capability proves invaluable in resource allocation, as those cryptogenic stroke patients deemed at a high risk of AF stand as prime candidates for extended cardiac monitoring, such as implantable loop recorders [21,31]. Recent findings from a randomized controlled trial further underscore the advantages of NT-proBNP, revealing that implantable loop-recorder screening correlates with a greater reduction in stroke risk among patients exhibiting higher NT-proBNP levels [31]. Ensuring thorough diagnostic investigation is imperative, given that detecting AF typically prompts a shift in secondary stroke-prevention strategy from antiplatelets to oral anticoagulants [19], known for their favorable efficacy and safety profile in AF patients [32]. A limitation of our meta-analysis is that there were inconsistencies in the timing of AF diagnosis post-enrolment in each study, and the duration of cardiac monitoring varied. The most common approach to AF detection was an ECG on admission and 24-h Holter monitoring; however, prolonged ECG monitoring outside the hospital was not always conducted. As a result, AF may have been underdiagnosed in some cases. Specific information on the AF detection strategies employed in each study is described in Table 1. In the era of reperfusion therapy, AF is an important clinical consideration as it mediates outcomes in acute ischemic stroke patients treated with intravenous thrombolysis [33]. This adds weight to the importance of NT-proBNP assessment, indicating that it may even be crucial in acutely prognosticating patients treated with reperfusion therapy.

Our study observed a higher diagnostic accuracy of NT-proBNP in patients with known etiology strokes than in those with cryptogenic strokes. This discrepancy likely stems from differences in AF subtypes between the two groups. Paroxysmal AF, known for its elusive detection compared to persistent or permanent AF [34], is presumed to be more prevalent in the cryptogenic stroke cohort than in patients with known etiology strokes [35]. Consequently, it is plausible that a larger proportion of patients in the cryptogenic stroke cohorts harbor undiagnosed paroxysmal AF. These patients may manifest as false positives in our analysis, potentially accounting for the comparatively lower specificity of 68%. To provide a more accurate assessment of NT-proBNP’s efficacy in cryptogenic stroke, future studies should extend the duration of cardiac monitoring to capture paroxysmal AF episodes better [36]. Moreover, a limitation lies in the variation in AF subtypes among different cohorts and inconsistent reporting of the number of patients with each subtype. Furthermore, the definition of cryptogenic stroke in each cohort (as described in Table 1) was not entirely consistent with the studies. Addressing these factors would enhance the precision and generalizability of our findings.

The elevation of NT-proBNP in AF patients primarily stems from increased stretching and stress on the myocardial wall [14]. Particularly, NT-proBNP levels are notably raised in AF patients with left atrial enlargement [37]. However, even in AF patients without left atrial enlargement, NT-proBNP levels remain higher than in non-AF patients, suggesting that the asynchronous myocardial stretches inherent in AF also contribute to NT-proBNP release [37]. Furthermore, NT-proBNP levels tend to decrease as the heart returns to sinus rhythm, resulting in lower levels in patients with paroxysmal AF compared to those with persistent or permanent AF [38]. This dynamic pattern reduces the effectiveness of NT-proBNP in cryptogenic stroke, where its primary potential lies in the risk stratification of patients with paroxysmal AF. A clinically significant entity is atrial cardiopathy, characterized by an elevated NT-proBNP and an increased stroke risk, often in the absence of AF [30,39]. In our study, these patients may have appeared as false positives, leading to an underestimation of specificity. Given that atrial cardiopathy frequently precedes AF, there remains uncertainty regarding the potential benefits of anticoagulation in these patients [30,39].

To improve the accuracy of NT-proBNP in clinical practice, researchers have explored several other biomarkers and clinical factors that can complement its diagnostic potential [30]. For instance, Kneihsl et al. [20] demonstrated that while NT-proBNP alone had a sensitivity of 58.3% in detecting AF following cryptogenic stroke, this sensitivity increased to 92% when integrating a risk score combining NT-proBNP with age and various indicators from brain imaging and echocardiography. Advanced machine learning algorithms may also aid in the prognostication of patients with suspected AF [40]. In addition to NT-proBNP, other biomarkers have been traditionally used; however, their efficacy in detecting AF has been variable and comparatively suboptimal for clinical use [41]. A detailed overview of these biomarkers is provided in Table 5.

Alongside blood-based biomarkers, growing evidence supports the role of clot morphology in determining the etiology of cryptogenic stroke [51,52,53]. Furthermore, standard cardiovascular laboratory parameters such as triglycerides and total cholesterol have also been shown to enhance the ability of NT-proBNP to distinguish between stroke of cardioembolic or large-artery atherosclerotic origin [54]. A recent prospective study also identified several biomarkers associated with AF in cryptogenic stroke patients [41]. Notably, NT-proBNP emerged as the robust predictor of AF at baseline and after 12-month follow-up [41]. Despite numerous proposals for tools or scores to assess the likelihood of AF post-stroke [17,20,55,56], a standardized system remains elusive. Similar to the CHA_2_DS_2_-VASc score’s role in guiding AF treatment [57], future research efforts could aim at developing a tool for AF detection. Our findings corroborate existing literature [20,39,41], suggesting that NT-proBNP could play a significant role in such a tool.

### Limitations

Our study had several limitations. Primarily, we identified only five studies meeting the inclusion criteria for our analysis on cryptogenic stroke [13,17,19,20,21] and four studies for the analysis on stroke of known etiology [13,18,19,22]. The main reason for this limitation was the necessity to exclude studies where we could not precisely determine the number of AF and non-AF patients with NT-proBNP levels above and below the cut-offs. Some of these studies reported rounded-off values for sensitivity and specificity [41,58], which were not compatible with the raw numbers required as inputs for the *midas* command in STATA. Additionally, one study utilized the lower and upper NT-proBNP quartiles as cut-offs to create high-sensitivity and high-specificity models instead of opting for a cut-off that balanced both [44].

The variations in NT-proBNP cut-offs emerged as a major limitation of this study. There were considerable variations in NT-proBNP cut-offs in our study, ranging from 250 pg/mL to 505 pg/mL (Table 3). Theoretically, a lower cut-off (such as 250 pg/mL) would yield higher sensitivity for AF diagnosis but lower specificity, and vice versa for a higher cut-off (such as 505 pg/mL). Unfortunately, there were insufficient studies at these data points for us to conduct subgroup analyses, and determining the ideal cut-off remains an area for future primary research. It is also possible that there is no single “ideal” cut-off, as clinicians may benefit from considering patient demographics and resource availability to decide if a higher-specificity or higher-sensitivity approach is more suitable for their circumstances. Nevertheless, our data suggest that any NT-proBNP value within the range of 250 to 505 pg/mL provides reasonable sensitivity and specificity for AF detection post-stroke.

While we initially planned to conduct a meta-analysis to evaluate differences in the mean NT-proBNP levels between AF and non-AF patients in these studies, the necessary data for this analysis were often unavailable and occasionally reported as the median and interquartile range without providing the first and third quartiles. Unfortunately, the software required mean and standard deviation values. Another limitation is that our study only utilized a single measurement of NT-proBNP rather than tracking changes over time. This is crucial, as the stroke itself may influence NT-proBNP levels due to the release of pro-inflammatory cytokines [30]. Lastly, our analysis focused on diagnostic accuracy. Therefore, while we suggested that NT-proBNP may be useful in stratifying patients for prolonged cardiac monitoring, further research is necessary to investigate which cardiac monitoring forms are most suitable for these patients, considering patient outcomes and resource allocation.

## 5. Conclusions

In conclusion, our study emphasizes the rapid increase in NT-proBNP concentration following the onset of atrial fibrillation, reinforcing the utility of this biomarker in early detection and diagnosis. Our meta-analysis found that NT-proBNP demonstrates good-to-very-good diagnostic accuracy in detecting AF following cryptogenic stroke, with excellent accuracy observed in patients with stroke of known etiology. This meta-analysis updates and consolidates the data to assess the diagnostic accuracy of NT-proBNP in detecting AF in the context of stroke. An integrated approach combining NT-proBNP measurements with ECG telemetry, clinical factors, and imaging tests like transthoracic and transesophageal echocardiography is critical in identifying the cause of stroke and enhancing diagnostic accuracy. Although consensus on a standardized risk tool is still pending, from a clinical perspective, NT-proBNP holds promise in identifying cryptogenic stroke patients who would benefit from extended cardiac monitoring. Further primary research is warranted to determine the optimal cut-off for NT-proBNP and to identify the most effective form of cardiac monitoring for these patients.

## Figures and Tables

**Table 1:** Baseline Characteristics of Studies Included in the Meta-analysis.

StudyID	Author	Year	Country	Study Type	Stroke Cohort	Number of Patients (n)	Male (*n*, %)	Age (Mean, SD)			AF Detection Method	Cryptogenic Stroke Diagnosis Method
								Overall	AF	Non-AF		
1	Amaya Pascasio et al. [17]	2022	Spain	Prospective ^a^	Cryptogenic stroke	460	308 (66.96)	65.0 (13.4)	74.7 (9.0)	62.0 (13.4)	Measured for at least 72 h with telemetry or Holter monitoring during hospitalization (and additional Holter monitoring was at the discretion of the neurologist). Additionally, a TTE was conducted during hospitalization or within 3 months.	Cryptogenic stroke was diagnosed where previously known AF, AF found at the emergency ECG, and other major cardioembolic causes were excluded. Patients with incomplete etiological investigations (e.g., missing TTE) were also excluded.
2	Fonseca et al. [18]	2011	Portugal	Prospective	Known etiology ^b^	66	40 (60.61)	-	63 (16)	59 (14)	At least two ECGs during hospitalization and 24-h Holter monitoring if the stroke cause remained undetermined. Additionally, TTE was performed in all patients, and TOE was performed in all patients aged < 55 years.	-
3a	Fonseca et al. [19]	2014	Portugal	Prospective	Known etiology	184	105 (57.07)	-	70.3 (7.8)	58.6 (12.7)	At least two ECGs and one 24 h Holter within the first week of presentation. Additionally, TTE was performed in all patients, and TOE was performed in all patients aged < 55 years.	-
3b	Fonseca et al. [19]	2014	Portugal	Prospective	Cryptogenic stroke	80	42 (52.50)	-	74 (7) ^c^	68 (15) ^c^	As stated above, with additional serial 24-h Holter monitoring conducted during follow-ups between 3 and 6 months.	Diagnosis of cryptogenic stroke was made using the TOAST criteria [27]. If a patient had a history of AF and no other findings were disclosed, then they were classified as cardioembolic rather than cryptogenic.
4a	Kneihsl et al. [13]	2019	Austria	Prospective	Known etiology	274	157 (57.30)	-	77.4 (9.5)	68.0 (12.6)	ECG on admission, ECG monitoring in the stroke unit, medical history. TTE was performed in all patients who did not have evidence of large- or small-vessel disease. TOE was performed in all patients aged < 55 or those with clinical and/or imaging signs of cardioembolism.	-
4b	Kneihsl et al. [13]	2019	Austria	Prospective	Cryptogenic stroke	143	77 (53.85)	-	74.4 (6.7)	66.9 (14.7)	As above, with additional stroke unit ECG monitoring of at least 48 h, regular pulse controls, and a further 24 h Holter monitoring during hospitalization.	Cryptogenic stroke was diagnosed by excluding patients with known AF or AF known immediately on admission, those with other known cardioembolic sources, those with large-vessel disease, patients with small-vessel disease, and those with specific stroke etiologies such as arterial dissection.
5	Kneihsl et al. [20]	2022	Austria	Prospective	Cryptogenic stroke	150	85 (56.67)	66.7 (15.3)	75.0 (6.3)	65.1 (15.9)	ECG on admission, continuous ECG in stroke unit, additional 24-h Holter monitoring, ECG during follow-up, and ILR offered to some patients at the discretion of the treating physicians. Additionally, echocardiography was performed for each patient.	Patients were classified as having cryptogenic stroke if there was no evidence of AF on initial ECG and 24-h Holter monitoring.
6	Rodriguez-Yanez et al. [21]	2013	Spain	Prospective	Cryptogenic stroke	264	150 (56.82)	-	81 (6)	72 (9)	ECG on admission and upon any arrhythmic pulse, 24 h Holter ECG in patients with a history of palpitations. In patients aged < 50, TOE and microbubble Doppler investigations were additionally conducted to detect right-to-left shunts.	Diagnosis of cryptogenic stroke was made using the TOAST criteria [27]. Cryptogenic stroke was diagnosed when no etiological sources were found. Cases with two co-existing causes of stroke were classified as undetermined rather than cryptogenic.
7	Zhao et al. [22]	2020	China	Retrospective	Known etiology	550 ^d^	236 (42.91)	71 (9)	72 (9)	71 (9)	ECG in all patients. In those with cardioembolic stroke, ECG, and 24-h Holter after admission. This was repeated within seven days in patients with STAF ≥5, where AF was still not found [28]. Additionally, either a TEE or TOE was conducted on each patient.	-

Abbreviations: AF = atrial fibrillation, n = number of patients, SD = standard deviation, ECG = electrocardiogram, ILR = implantable loop recorder, STAF = score for the targeting of atrial fibrillation, TTE = transthoracic echocardiogram, TOE = transesophageal echocardiogram, TOAST = Trial of Org 10172 in Acute Stroke Treatment. ^a^ This was a retrospective analysis of a prospectively collected dataset. ^b^ This study was used in the stroke of known etiology meta-analysis since it classified the stroke etiology as cardioembolic or non-cardioembolic. ^c^ This study reported the ages using the median (interquartile range) for these cohorts. ^d^ This study matched AF and non-AF patients.

**Table 2:** Prevalence Rates of Comorbidities among Studies Included in the Meta-analysis.

Study ID	Author	Year	Stroke Cohort	Number of Patients (n)	Hypertension (n, %)	Diabetes (n, %)	Dyslipidemia (n, %)	Smoking (n, %)
1	Amaya Pascasio et al. [17]	2022	Cryptogenic stroke	460	276 (60.00)	114 (24.78)	191 (41.52)	240 (52.17)
2	Fonseca et al. [18]	2011	Known etiology	66	33 (50.00)	8 (12.12)	20 (30.30)	26 (39.39)
3a	Fonseca et al. [19]	2014	Known etiology	184	127 (69.02)	47 (25.54)	83 (45.11)	43 (23.37)
3b	Fonseca et al. [19]	2014	Cryptogenic stroke	80	57 (71.25)	23 (28.75)	26 (32.50)	7 (8.75)
4a	Kneihsl et al. [13]	2019	Known etiology	274	236 (86.13)	35 (12.77)	162 (59.12)	86 (31.39)
4b	Kneihsl et al. [13]	2019	Cryptogenic stroke	143	111 (77.62)	30 (20.98)	71 (49.65)	40 (27.97)
5	Kneihsl et al. [20]	2022	Cryptogenic stroke	150	100 (66.67)	24 (16.00)	72 (48.00)	36 (24.00)
6	Rodriguez-Yanez et al. [21]	2013	Cryptogenic stroke	264	138 (52.27)	68 (25.76)	78 (29.55)	53 (20.08)
7	Zhao et al. [22]	2020	Known etiology	550	417 (75.82)	119 (21.64)	-	75 (13.64)

Abbreviation: n = number of patients.

**Table 3:** Sensitivity and Specificity of NT-proBNP in Detecting AF within Each Individual Study Cohort.

Study ID	Author	Year	Stroke Cohort	Sensitivity (%)	Specificity (%)	NT-proBNP Cut-Off (pg/mL)
1	Amaya Pascasio et al. [17]	2022	Cryptogenic stroke	81/102 (79.4)	247/358 (69.0)	250
2	Fonseca et al. [18]	2011	Known etiology	17/18 (94.4)	35/48 (72.9)	265.5
3a	Fonseca et al. [19]	2014	Known etiology	55/55 (100.0)	91/129 (70.5)	265.5
3b	Fonseca et al. [19]	2014	Cryptogenic stroke	15/17 (88.2)	39/63 (61.9)	265.5
4a	Kneihsl et al. [13]	2019	Known etiology	96/103 (93.2)	123/171 (71.9)	505
4b	Kneihsl et al. [13]	2019	Cryptogenic stroke	12/14 (85.7)	84/129 (65.1)	505
5	Kneihsl et al. [20]	2022	Cryptogenic stroke	14/24 (58.3)	104/126 (82.5)	505
6	Rodriguez-Yanez et al. [21]	2013	Cryptogenic stroke	13/15 (86.7)	146/249 (58.6)	360
7	Zhao et al. [22]	2020	Known etiology	252/275 (91.6)	222/275 (80.7)	431

Abbreviations: NT-proBNP = N-terminal pro-B-type natriuretic peptide.

**Table 4:** Meta-analysis Summary of NT-proBNP Diagnostic Accuracy in AF Detection Post-stroke.

	Cryptogenic Stroke (95% CI)	Stroke of Known Etiology (95% CI)
Number of studies	5	4
Number of patients	1097	1074
AUROC	0.80 (0.76–0.83)	0.91 (0.88–0.93)
Sensitivity	0.81 (0.68–0.89)	0.95 (0.90–0.97)
Specificity	0.68 (0.60–0.75)	0.75 (0.69–0.80)
PLR	2.5 (2.1–3.0)	3.7 (3.1–4.5)
NLR	0.28 (0.18–0.45)	0.07 (0.04–0.14)
Diagnostic odds ratio	9 (6–14)	52 (27–98)
Pre-test probability of disease	172/1097 ≈ 0.16	451/1074 ≈ 0.42
Deviance	57.5	43.1
AIC	67.5	53.1
BIC	69.0	53.5
BICdiff	9.7	−2.6
Correlation (mixed model)	−1.00	−1.00
Proportion of heterogeneity likely due to the threshold effect	1.00	1.00
ICC_SEN	0.09 (0.00–0.25)	0.04 (0.00–0.16)
MED_SEN	0.63 (0.55–0.82)	0.58 (0.51–0.88)
ICC_SPE	0.04 (0.00–0.10)	0.01 (0.00–0.04)
MED_SPE	0.59 (0.54–0.69)	0.55 (0.51–0.65)
Heterogeneity (I^2^)	Sensitivity: 47.99 (0.00–100.00)	Sensitivity: 40.67 (0.00–100.00)
	Specificity: 83.02 (69.03–97.01)	Specificity: 57.99 (11.72–100.00)

Abbreviations: NT-proBNP = N-terminal pro-B-type natriuretic peptide, CI = confidence interval, AUROC = area under receiver operating characteristic curve, PLR = positive likelihood ratio, NLR = negative likelihood ratio, AIC = Akaike information criteria, BIC = Bayesian information criteria, ICC_SEN = interstudy variation in sensitivity, MED_SEN = interstudy variation in sensitivity, ICC_SPE = interstudy variation in specificity, MED_SPE = interstudy variation in specificity, I^2^ = percentage of variation among studies due to heterogeneity.

**Table 5:** Various Biomarkers and their Diagnostic Accuracy, Sensitivity, and Specificity in Identifying Atrial Fibrillation Following Cryptogenic Stroke.

Biomarker	Definition in the Context of AF	Diagnostic Accuracy	Sensitivity	Specificity
NT-proBNP	A prohormone that is released in response to myocardial stress and volume overload, which may be more likely to occur in patients with AF [14,15].	Our investigation revealed a good-to-very-good diagnostic accuracy of NT-proBNP for detecting AF in cryptogenic stroke patients, with an AUROC of 0.80 [95% CI 0.76–0.83].	Our findings show NT-proBNP has a very good sensitivity of 0.81 [95% CI 0.68–0.89] for detecting AF in cryptogenic stroke patients.	Our study demonstrated that NT-proBNP has a sufficient specificity of 0.68 [95% CI 0.60–0.75] for detecting AF in cryptogenic stroke patients.
Cardiac troponins	These cardiac enzymes are involved in myocyte contractility and may be released in greater quantities in AF patients due to myocyte damage and transient ischemia [42].	A recent analysis reported an AUROC of 0.697 [95% CI 0.551–0.842] for hs-cTnT and an AUROC of 0.65 [95% CI 0.556–0.743] for hs-cTnI for detecting AF in cryptogenic stroke and TIA patients [41]. Comparatively, NT-proBNP had a greater AUROC of 0.725 [95% CI 0.642–0.808].	Troponin elevations are a predictor of AF in cryptogenic stroke [43,44]; however, there is limited data on their sensitivity in this context.	Whilst troponins are associated with AF in cryptogenic stroke patients [43,44], there are limited data on their exact specificity.
D-dimer	A product of the degradation of fibrin, typically used in venous thromboembolism, may also be increased in patients with AF [45].	A recent analysis reported an AUROC of 0.584 [95% CI 0.495–0.672] for detecting AF in cryptogenic stroke and TIA patients [41].	Limited data with various indications.	Limited data with various indications.
CRP	CRP is a biomarker that reflects systemic inflammation, which may reflect underlying AF, though it is generally non-specific [46].	Whilst cumulative exposure to CRP has been linked to AF, baseline CRP was shown not to be significantly associated with AF [46]. Moreover, there is a lack of data regarding the accuracy of CRP for diagnosing AF in cryptogenic stroke patients.	Limited data with various indications.	Limited data with various indications.
Galectin-3	Galectin-3 is a biomarker associated with myocardial fibrosis, which can occur due to structural remodeling in AF [47].	A prospective study reported that a galactin-3 level ≥ 9 ng/mL had a very good AUROC of 0.829 [95% CI 0.764–0.894] for detecting AF in stroke patients without previously known AF [48]. In the same study, NT-proBNP had a very good AUROC of 0.879 [95% CI 0.818–0.940].	Limited data with various indications.	Limited data with various indications.
Markers of oxidative stress (MDA, 8-isoprostane)	Oxidative stress is a factor involved in the pathogenesis of AF, and there are numerous biomarkers of oxidative stress that may be elevated in AF patients [49].	Whilst MDA activity is associated with AF [50], there are limited data on the diagnostic accuracy of oxidative stress biomarkers in the setting of cryptogenic stroke.	Limited data with various indications.	Limited data with various indications.

Abbreviations: AF = atrial fibrillation, NT-proBNP = N-terminal pro-B-type natriuretic peptide, CI = confidence interval, AUROC = area under receiver operating characteristic curve, hs-cTnT = high-sensitivity cardiac troponin T, hs-cTnI = high-sensitivity cardiac troponin I, TIA = transient ischemic attack, CRP = C-reactive protein.

## Data Availability

The original contributions presented in the study are included in the article and online Supplementary Information, and further inquiries can be directed to the corresponding author.

## Supplementary Materials

The following supporting information can be downloaded at: https://www.mdpi.com/article/10.3390/pathophysiology31030024/s1, Figure S1: Summary receiver operating character (SROC) for A (patients with cryptogenic stroke) and B (patients with stroke of known etiology); Figure S2: Likelihood ratio matrix for A (patients with cryptogenic stroke) and B (patients with stroke of known etiology); Figure S3: Goodness of fit for A (patients with cryptogenic stroke) and B (patients with stroke of known etiology); Table S1: Preferred Reporting Items for Systematic Reviews and Meta-Analyses (PRISMA) 2020 checklist; Table S2: Meta-analysis of Observational Studies in Epidemiology (MOOSE) checklist; Table S3: STARD-2015 Checklist for Diagnostic Accuracy Studies; Table S4: Methodological quality assessment of included studies using the modified Jadad scale and assessment of funding bias.

## Abbreviations

AF	Atrial fibrillation
ECG	Electrocardiography
ICM	Implantable cardiac monitor
NT-proBNP	N-terminal pro-B-type natriuretic peptide
BNP	B-type natriuretic peptide
RAAS	Renin-angiotensin-aldosterone system
AUROC	Area Under the Receiver Operating Curve
PRISMA	Preferred Reporting Items for Systematic Reviews and Meta-Analyses
MOOSE	Meta-analysis of Observational Studies in Epidemiology
STARD	Standards for Reporting Diagnostic Accuracy Studies
SROC	Summary receiver operating characteristic
